# Gut microbiome in intracranial aneurysm growth, subarachnoid hemorrhage, and cerebral vasospasm: a systematic review with a narrative synthesis

**DOI:** 10.3389/fnins.2023.1247151

**Published:** 2023-10-19

**Authors:** Tomasz Klepinowski, Karolina Skonieczna-Żydecka, Bartłomiej Pala, Ewa Stachowska, Leszek Sagan

**Affiliations:** ^1^Department of Neurosurgery, Pomeranian Medical University, Szczecin, Poland; ^2^Department of Biochemical Sciences, Pomeranian Medical University, Szczecin, Poland; ^3^Department of Human Nutrition and Metabolomics, Pomeranian Medical University, Szczecin, Poland

**Keywords:** aneurysm, hemorrhage, vasospasm, brain, microbiome

## Abstract

Intracranial aneurysms (IA) are the most common cerebral vascular pathologies. Their rupture leads to the most dangerous subtype of stroke—aneurysmal subarachnoid hemorrhage (aSAH), which may be followed by cerebral vasospasm and ischemic sequelae. Recently, an imbalance within the intestinal microbiota, referred to as dysbiosis, was suggested to play a role in the formation, progression, and rupture of IA. As no systematic review on this topic exists, considering the significance of this matter and a lack of effective prophylaxis against IA or cerebral vasospasm, we aim to sum up the current knowledge regarding their associations with intestinal microbiome, identify the gaps, and determine future prospects. Scientific databases were systematically and independently searched by two authors from inception to 1st May 2023 for original articles regarding the role of intestinal microbiota in intracranial aneurysmal growth, aSAH occurrence, as well as in cerebral vasospasm following aSAH. The PRISMA (Preferred Reporting Items for Systematic Reviews and Meta-Analyses) checklist was followed in an abstraction process. The STROBE tool was applied to assess the risk of bias. This research was funded by the National Science Centre, Poland (grant number 2021/41/N/NZ2/00844). Of 302 records, four studies were included that fully met eligibility criteria. Studies reported (1) that the relative abundance of *Hungatella hathewayi* is a protective factor against aneurysm growth and rupture, resulting from the reduced inflammation and extracellular matrix remodeling in the cerebral arterial wall and from reduced metalloproteinase-mediated degradation of smooth muscle cells in cerebral vessels. (2) Relative abundance of *Campylobacter ureolyticus* is associated with aSAH. (3) No article has evaluated microbiota in relation to cerebral vasospasm following aSAH although there is an ongoing study. We concluded that intestinal microbiota might be a potential target for diagnostic and therapeutic tools to improve the management of cerebral aneurysms. However, more studies of prospective design are needed.

## Cerebral aneurysm and intestinal microbiome

Cerebral aneurysms are the most common intracranial vascular pathologies and are found in approximately 3% of the global population (Vlak et al., 2011). Their rupture leads to the most dangerous subtype of stroke, aneurysmal subarachnoid hemorrhage (aSAH), affecting up to 450,000 people worldwide per year (Etminan et al., 2019). Such a hemorrhage can be a devastating cerebrovascular incident as 10–15% of subjects die immediately at the scene of the event (Greenberg, 2019). Those who survive the initial ictus are at risk of various complications. One of the most dangerous and dreaded sequelae is cerebral vasospasm and delayed cerebral ischemia. Roughly half of the patients experience vascular spasm peaking on post-bleeding days 7 to 8, which kills up to 10% of the remaining survivors, whereas another 10% is left disabled (Greenberg, 2019).

The symbiotic intestinal microbiome modulates the host's health via several regulatory routes, so-called axes, all of which constitute immune-related pathways. One of such is the gut–microbiota–brain axis (GMBA). The GMBA is the physical and functional connection between the central nervous system (CNS) and the gastrointestinal tract. Structurally and chemically, the GMBA includes the vagus nerve, spinal dorsal root ganglia, the autonomic nervous system of the gut, and numerous biochemical and immune pathways. Bidirectional signaling in the axis takes place through hormones, cytokines, and bacterial metabolites secreted in the intestinal lumen. The enteric nervous system regulates, among other things, intestinal peristalsis and ensures adequate digestion and fermentation of food. This system also plays an important role in regulating hunger and satiety as well as the perception of pain in the abdominal cavity. On the other hand, it has been proven that neuroactive and immunocompetent metabolites of the microbiota are essential for shaping the structure and function of key areas of the brain, especially the limbic system, involved in emotions (Cryan et al., 2019). Moreover, via the metabolic activity of the microbiota, neuronal processes and plasticity are regulated, including the transmission of neural signals and the expression of neurotrophins (Cryan et al., 2019).

An imbalance within the intestinal microecological niche, referred to as dysbiosis, has been shown to constitute a risk factor for a number of pathologic conditions including ischemic stroke (Pluta et al., 2021), schizophrenia (Ghorbani et al., 2021; Samochowiec and Misiak, 2021), major depression (Simpson et al., 2021), abdominal aortic aneurysm (Tian et al., 2022), Takayasu arteritis (Desbois et al., 2021), coronary heart disease (Liu H. et al., 2020), and others (Hou et al., 2022). The intestinal microbiome affects blood vessels by means of bidirectional interactions with its endothelium and when dysbiosis occurs, and it might be associated with endothelial dysfunction through a number of mechanisms such as an increase in angiotensin-converting enzyme activity and promotion of inflammatory response as well as oxidative stress (Leslie and Annex, 2018). Clinically, endothelial dysfunction usually presents as hypertension but is also a contributing factor to atherosclerosis and diabetic peripheral arterial disease (Li et al., 2017; Biscetti et al., 2019). However, not until 2019 was it no man's land in terms of associations between intestinal microbiota and cerebral aneurysms. Finally, the increasing appreciation of the gut–brain–microbiota axis and its influence on the cardiovascular system led researchers to investigate the hypothesis of whether there exists an association between intestinal microbiota and intracranial aneurysms (Shikata et al., 2019). Since then, important steps have been taken toward understanding the metagenetic fundamentals of growth, progression, and rupture of intracranial aneurysms (IA).

Therefore, considering the significance of this matter and the lack of effective prophylaxis against IA and cerebral vasospasm, a timely systematic review is of utmost relevance to pinpoint the current knowledge, identify the gaps, and determine future prospects. This study aimed to answer research questions of what characteristics of intestinal microbiota are associated with the development and rupture of intracranial aneurysms and with cerebral vasospasm following aSAH. To comprehensively present the topic, our review shared the methodology of systematic review.

## Methods

Two researchers (TK and BP) independently searched major databases (PubMed MEDLINE, Scopus, and Web of Science) for articles pertaining to both (1) gut microbiota and (2) intracranial aneurysms or aSAH or cerebral vasospasm after aSAH. The following MeSH (Medical Subject Headings) and non-MeSH terms were used: “intracranial aneurysm,” “cerebral vasospasm,” “subarachnoid hemorrhage,” “microbiota,” “brain–gut axis,” and “metagenetics” with corresponding synonyms. Additional records were identified via hand search through references and citations of available reviews. The full electronic search strategy can be seen in Supplementary material 1. All languages were acceptable. The databases were searched in two waves, on 1 October 2022 and on 1 May 2023, followed by a de-duplication process (removing duplicate findings of the two initial independent searches). The PRISMA (Preferred Reporting Items for Systematic Reviews and Meta-Analyses) checklist was adhered to—see Supplementary material 2 (Liberati et al., 2009). Next, the publications were subject to eligibility criteria.

We included only original peer-reviewed studies that reported relations between intestinal microbiota and intracranial aneurysms or aSAH or cerebral vasospasm after aSAH. Both human and animal studies were acceptable. The exclusion criteria were as follows: (1) extracranial aneurysms, (2) letters to editors, (3) reviews, and (4) short responses. Eligibility was evaluated by TK and BP In case of disagreement, consensus was reached by consulting with a senior researcher (LS). Once the eligible articles were gathered, data extraction was performed.

The eligible publications were perused in detail and the following items were extracted: (1) study design, (2) timing and method of microbiota examination, (3) subject characteristics, (4) microbiota characteristics, (5) microbiota analysis technique, (6) metabolomic analyses, (7) relations to intracranial aneurysm growth, subarachnoid hemorrhage, or cerebral vasospasm following aSAH—comprising the phenotype manifestation of gathered microbiota data. Next, the extracted pieces of information were curated in the spreadsheet of Excel Microsoft 365 version 2303 (Redmond, USA), which can be accessed as Supplementary material 1.

To address quality and the risk of bias within the individual articles, we checked whether they conformed to the Strengthening the Reporting of Observational Studies in Epidemiology (STROBE) statement. This tool consists of 22 items that guide appropriate reporting in observational studies. As arbitrarily accepted, if the number of points was below 11 (50% of the maximum STROBE score), then such a study was deemed to be of low quality. If an article addressed between 11 and 14 items, moderate quality was attributed. In case of 15–18 or more than 18 points, then high or very high quality was assigned, respectively.

Extracted data underwent narrative synthesis, for which three rounds of the Delphi procedure were performed to reach a consensus. Faculty expertise was provided by expert professors in the field of microbiomics (KS-Ż and ES) and an expert professor in neurosurgery (LS).

## Gut–brain axis alterations might confer risk toward cerebral aneurysm

**Figure 1:**
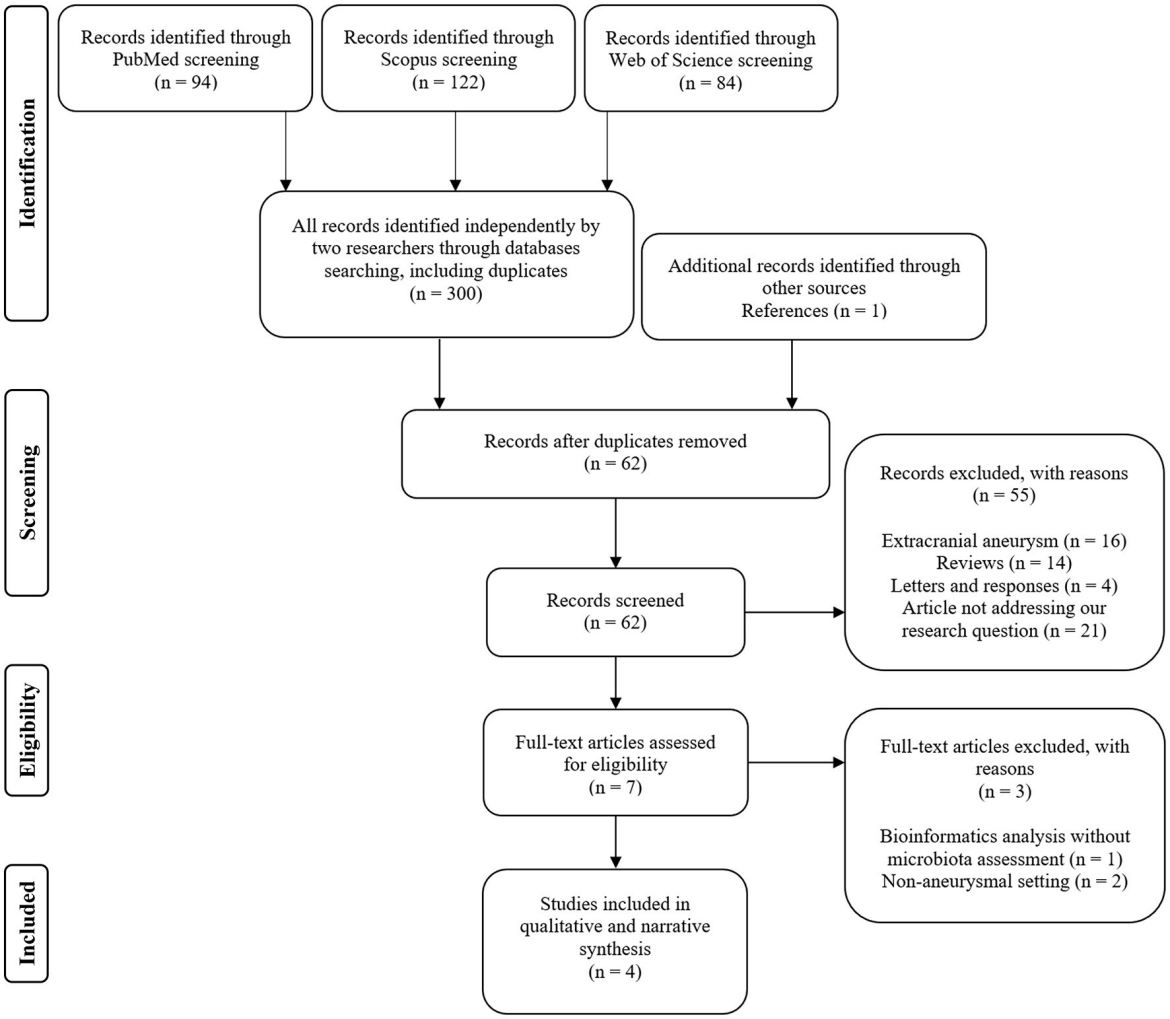
Flow diagram illustrating the selection process and reasons for exclusion.

Two independent searches of three major databases (PubMed, Scopus, and Web of Science) generated a total of 300 records (including duplications) and one additional through references. After the de-duplication process, 62 abstracts were evaluated for eligibility criteria. Subsequently, the curation of seven full texts allowed for narrowing down the results to four eligible studies that met the inclusion criteria—see Figure 1 (Shikata et al., 2019; Li et al., 2020; Kawabata et al., 2022; Sun et al., 2022). Of these, three publications sought the relationship between intestinal microbiota and the growth of intracranial aneurysms (Shikata et al., 2019; Li et al., 2020; Sun et al., 2022). Two articles evaluated microbiome in subjects with subarachnoid hemorrhage due to rupture of the aneurysm (Li et al., 2020; Kawabata et al., 2022). In terms of geographic distribution, three studies were from Asia and one from North America. Observational case–control study design prevailed (see Table 1 and Supplementary material 1). Although two studies (Rustia et al., 2021; Xiao et al., 2022) addressed cerebral ischemic complications due to endothelial dysfunction or cerebral hypoperfusion in subjects with altered microbiota, they were not in the setting of aneurysmal rupture. Therefore, they were not included in the systematic review but were discussed as a pathophysiological rationale for conducting a research study on associations between cerebral vasospasm and intestinal microbiome (see Cerebral vasospasm and delayed cerebral ischemia Section). Two studies were of high-quality based on the STROBE statement score and two studies were of very high quality. The most common shortcoming of quality is sample size estimation. Table 1 collectively presents the assessment of quality and the risk of bias. A full assessment of each study based on the STROBE statement can be viewed as Supplementary material 3.

**Table 1:** General characteristics of the included studies.

**References**	**Region**	**Design**	**Model**	**Outcome**	**STROBE score**	Quality^*^
Shikata et al. (2019)	North America	*In vivo* longitudinal interventional	Animal	Growth of UIA	17/22	High
Li et al. (2020)	Asia	Case–control observational (a) and *in vivo* (b)	Human (a) and animal (b)	Growth of UIA and aSAH	18/22	High
Sun et al. (2022)	Asia	Case–control observational	Human	Symptomatic progression of UIA	19/22	Very high
Kawabata et al. (2022)	Asia	Case–control observational	Human	aSAH	20/22	Very high

The STROBE score refers to the number of STROBE statement items that were addressed in a given study. Quality^*^: low if the STROBE score was < 11; moderate if the STROBE score was 11–14; high if the STROBE score was 15–18; very high if the STROBE score was 19–22. aSAH, aneurysmal subarachnoid hemorrhage; STROBE, Strengthening the Reporting of Observational Studies in Epidemiology; UIA, unruptured intracranial aneurysm.

## Aneurysm formation and growth

Three studies reported an association between dysbiosis of gut microbiota and aneurysm progression (Shikata et al., 2019; Li et al., 2020; Sun et al., 2022). Shikata et al. (2019) presented an experimental animal model of aneurysm formation in mice whose gut microbiota was antibiotically depleted. Depletion of gut microbiota was obtained by a 3-week feeding regimen with an antibiotic cocktail consisting of vancomycin, metronidazole, neomycin, and ampicillin. A control group had intact intestinal microbiota. The formation of the intracranial aneurysms was initiated by the introduction of elastase into subarachnoid cisterns in the region of the circle of Willis. The authors noticed a statistically significant risk reduction of aneurysm formation in subjects with depleted gut microbiota (6 vs. 83%; *p* < 0.001). Furthermore, microbiota-depleted mice had a higher symptom-free rate, which was portrayed in the Kaplan–Meier curve (76 vs. 6% at 21 days of the experiment; *p* < 0.001). Their results remained statistically significant after sensitivity analysis. For the quantitative assessment of the inflammatory process within the arterial wall of aneurysms, the authors compared mRNA expression of inflammatory molecules such as IL-1β, IL-6, and iNOS, as well as CD-68-positive macrophage counts in both groups (microbiota-depleted vs. controls). Those with depletion demonstrated significantly reduced mRNA expression of the inflammatory cytokines and lesser macrophage infiltration than mice with intact gut microbiota. It was the first study to investigate relations between cerebral aneurysms and intestinal microbiome, reverberating with a domino effect of new studies in the following years.

Subsequently, Li et al. (2020) published a quality study of meticulously conducted case–control study, in which they indicated that gut microbiota might play a role in the development and growth progression of intracranial aneurysms also in human beings (Li et al., 2020). The authors compared stool samples of 100 subjects with unruptured aneurysm with 100 controls without intracranial aneurysms. Alfa-diversity expressed that the Shannon index did not differ between the groups (*p* = 0.248), whereas beta-diversity, specified as Bray–Curtis dissimilarity, was statistically higher in controls (*p* = 0.001). The study group with unruptured aneurysms was found to have lower relative abundances of *H. hathewayi*. They confirmed the results in both the discovery and validation phases. Moreover, this bacterial abundance was positively correlated with circulating taurine levels (Spearman's rank correlation; rho ~ 0.8; *p* < 0.05). In order to search for a causal role of *H. hathewayi* in the development of intracranial aneurysms, additional analysis of the mouse model was performed. Stool transplantation was conducted from IA to control patients. The researchers found 14 differentially abundant species between mice treated with UIA patient feces and those treated with control feces. The differentially abundant species included *H. hathewayi, O. splanchnicus, A. putredinis*, and *B. intestinalis*. After IA induction with the use of elastase, mice receiving IA patients-derived fecal transplants had a significantly increased overall incidence of aneurysms and a significant increase in aneurysmal rupture. Fecal microbiota transplantation (FMT) from subjects with IA-induced overrepresentation of inflammatory processes, cell adhesion, response to oxidative stress, and the extracellular matrix as evidenced by the analyses of the transcriptional profile of cerebral vessels. FMT from patients with IA also diminished the production of taurine, L-histidine, and linoleic acid. Furthermore, feeding with *H. hathewayi* gavage decreased the incidence of intracranial aneurysms after elastase exposure (45% with gavage vs. 90% without gavage; *p* = 0.006). Additionally, *H. hathewayi* gavage reduced rupture rate (44 vs. 83%; *p* = 0.026), also in Kaplan–Meier analysis (*p* = 0.0134). Similar results were obtained with taurine supplementation. Overall, the proper abundance of *H. hathewayi* in gut microbiota might protect against intracranial aneurysms (see Table 2).

**Table 2:** Detailed characteristics of the included studies.

**References**	**Clinical phenotype**	**Subjects (*n*, age, gender)**	**Treatment protocol (type, dose, and duration)**	**Microbiota analyses details**	**Main microbiota results**	**Microbiota-associated phenotype**	**Concluding remarks**
Sun et al. (2022)	Unruptured intracranial aneurysms: symptomatic (sUIA) and asymptomatic (aUIA)	32 patients (aUIA: *n* = 86; sUIA: *n* = 46); 55.86 ± 11.33 y.o.; 94 females	Non-interventional	16S sequencing (V3–V4 region); rarefaction to 30,000 reads; NGS in Illumina NovaSeq platform	- Seven bacterial taxa discriminating aUIA and sUIA; - Symptomatic UIA: ↑*Ruminococcaceae* (genera: F*ournierella, Ruthenibacterium*, and *Anaerotruncus*), *Clostridiales*, ↓*Fusobacteria*;	- sUIA: 19 functional orthologs changed when compared with aUIA - sUIA: ↓propionate synthesis; ↑ peptidoglycan biosynthesis, expression of chromosome associated protein, transcription machinery, DNA replication proteins, ribosome biogenesis, RNA biogenesis	Microbiota might discriminate symptoms in UIA
Liu H. J. et al. (2020)	Unruptured intracranial aneurysms (UIA)	140 UIA patients and 140 age-, sex- and blood pressure-matched controls; ND., ND.	Non-interventional	Insert size 350 bp; read length 150 bp; Illumina HiSeq X Ten platform	- UIA patients: ↑*Bacteroides, Parabacteroides, Ruminococcus*, and *Blautia* were significantly, ↓*Faecalibacterium, Eubacterium, Collinsella*, and *Lactobacillus;* - Forty-seven differentially abundant bacteria might serve as UIA prognostic factor (AUC of up to 0.86) and a 95% confidence interval (CI) of 0.81–0.91	- Forty-six metabolic pathways differentially abundant between UIA patients and controls; - UIA: ↑ unsaturated fatty acid biosynthesis; ↓ biosynthesis of threonine, isoleucine, lysine, and methionine	*H. hathewayi* and associated taurine depletion might be a key factor in the pathogenesis and clinical course of IAs
Liu H. et al. (2020)	Unruptured intracranial aneurysms (UIA)	Wild-type C57BL/6N mice; 10-week males	- A depletion of git microbiota with a broad-(1 g/L ampicillin, 1 g/L metronidazole, 1 g/L neomycin, and 0.5 g/L vancomycin) via autoclaved (tap) drinking water for 2 weeks—IUA formation: right renal artery and the left common carotid artery of mice ligation and elastase injection; - Fresh fecal samples of the UIA and controls transplanted twice at a 1-day interval via gavage followed by taurine at 150 mg/day, L-histidine at 500 mg/day, or linoleic acid at 20 mg/day supply; - Administration of *H. hathewayi* (1 × 10^9^ CFU/mouse in sterile PBS) or sterile PBS, three times a week	ND	- Fourteen differentially abundant species between mice treated with UIA patient feces and those treated with control feces; - Mice transplanted with UIA feces: ↓*H. hathewayi, O. splanchnicus, A. putredinis, B. intestinalis, and B. nordii*	- Mice receiving IA patients fecal transplant:↑ the overall incidence of aneurysms and its rupture; - UAI gut transplant mice: ↑ inflammatory processes, cell adhesion, response to oxidative stress, and the extracellular in cerebral vessels; ↓ production of taurine, L-histidine, and linoleic acid; - Taurine and *H. hatheway* supplementation ↓the overall incidence of aneurysms and its rupture rates	
Kawabata et al. (2022)	Unruptured intracranial aneurysms (UIAs) and ruptured aneurysms (RA)	61 patients; 67.35 ± 11.42 y.o.; 25 females	Non-interventional	16S sequencing (V1–V2 region); 250 bp paired-end; rarefaction to 26 830 reads; NGS in Illumina MiSeq platform; 500 Cycles	- No difference in alpha diversity and richness between RA and UIAs and irrespective of IA grade; - Significant difference in beta-diversity; - RA: ↑*Campylobacteriota (*species *C. ureolyticus, C. concisus, C. gracilis, and C. hominis)*;	ND	The genus *Campylobacter* and genus *Campylobacter ureolyticu*s may be associated with the rupture of IA
Shikata et al. (2019)	IA formation: systemic hypertension and a single injection of elastase into the cerebrospinal fluid	10- to 12-week-old C57BL/6J mice; ND	- Microbiota depletion: antibiotic cocktail composed of vancomycin (0.5 g/L), metronidazole (1 g/L), ampicillin (1 g/L), and neomycin (1 g/L); 3 weeks prior to IA formation until 1 day before; - IA formation: systemic hypertension and a single injection of elastase into the cerebrospinal fluid	ND	Mice with depleted gut microbiota: ↓ incidence of aneurysms and its rupture, ↑ symptom-free rate	Mice with depleted gut microbes: ↓ macrophages (CD68-positive cells) in the aneurysmal walls (4.0 ± 2.3 vs. 9.4 ± 6.4/0.01 mm^2^); ↓ IL-6, IL-1B and iNOS	Gut microbiota might contribute to IA formation via inflammation

CI, confidence interval; IA, intracranial aneurysm; UIA, unruptured intracranial aneurysm; sUIA and aUIA, symptomatic and asymptomatic unruptured intracranial aneurysms; RA, ruptured aneurysm; ND, not determined; ↓, decreased; ↑, increased.

## Aneurysmal subarachnoid hemorrhage

Two research articles (Li et al., 2020; Kawabata et al., 2022) addressed the question of whether changes in the gut microbiome could be associated with rupture of the aneurysm and subsequently with aneurysmal subarachnoid hemorrhage. As demonstrated before (Li et al., 2020), the counts of *H. hathewayi* might confer protection against aneurysmal rupture. Kawabata et al. (2022) in their case–control study compared the microbiota of subjects with aSAH (*n* = 28) to those with unruptured intracranial aneurysms (*n* = 33). Patients' stool samples were collected within 48 h after admission and a gene coding 16S rRNA was sequenced. At the phylum level, the authors report an intestinal abundance of Campylobacteriota (*q* = 0.007). At the genus level, the abundance of *Campylobacter* spp. in the aSAH group was elevated as compared to the UIA group (*q* = 0.005 in 16S rRNA sequencing and *q* = 0.0012 in PCR). Of four *Campylobacter species* detected in a phylogenetic tree (*C. ureolyticus, Campylobacter concisus, Campylobacter gracilis*, and *Campylobacter hominis*), only *C. ureolyticus* was significantly more abundant in gut microbiota among aSAH subjects in both PCR analysis and 16S rRNA sequencing (*q* = 0.0016 and *q* = 0.028, respectively). No taxa were found with reference to the severity of subarachnoid hemorrhage defined as low (Hunt-Hess I–III) or high-grade aSAH (Hunt-Hess IV–V). Hence, aneurysmal subarachnoid hemorrhage is associated with the abundance of Campylobacter spp., but it is unclear whether the causal relationship exists, or it is merely an epiphenomenon.

## Symptomatic unruptured intracranial aneurysms

The instability of the aneurysmal wall precedes rupture and might present as sentinel headaches, oculomotor nerve palsy, or beating aneurysm sign in computed tomography angiograms (Greenberg, 2019). It is of high importance to promptly identify those patients because they are at the highest risk of rupture and aneurysmal subarachnoid hemorrhage. To date, there has been only one study comparing the gut microbiota of symptomatic UIA subjects with asymptomatic UIA. Sun et al. (2022) reported results of a study comparing 46 symptomatic UIA and 86 asymptomatic UIA, excluding any patients with a history of aSAH. Fecal samples were collected upon admission, and DNA was extracted and sequenced using the Illumina NovaSeq platform (San Diego, CA, United States)—a summary of the methodology is listed in Supplementary material 1. There were no differences in alpha diversity between the groups. On the other hand, beta diversity (Bray-Curtis distance) revealed significant differences in gut taxonomic composition (*p* = 0.038). Firmicutes at the phylum level and Prevotellaceae with Ruminococcaceae at the family level were relatively abundant in the symptomatic cohort. Furthermore, *Lawsonibacter* positively correlated whereas *Burkholderia* and Ruminiclostridium negatively correlated with the size of the aneurysms (*p* < 0.05 and *p* < 0.01, respectively).

Importantly, in symptomatic patients, *Fusobacteria* counts were diminished. Functional analysis demonstrated that there were 19 functional orthologs altered when compared with asymptomatic counterparts. These were pathways linked to lowered propionate synthesis and elevated peptidoglycan biosynthesis along with elevated production of proteins taking part in expression processes. Overall, these findings suggest there is a specific metagenomic signature of gut microbiota in subjects with symptomatic and potentially unstable intracranial aneurysms at a higher risk of rupture.

## Cerebral vasospasm and delayed cerebral ischemia

To date, there has been no study seeking a relationship between an altered gut microbiota and cerebral vasospasm. Thus, the authors of this review have commenced the first such research to aid the body of neurosurgical and microbiomic literature (see Future prospects Section). The rationale for such studies is discussed below.

## Diagnostic and therapeutic potential

**Figure 2:**
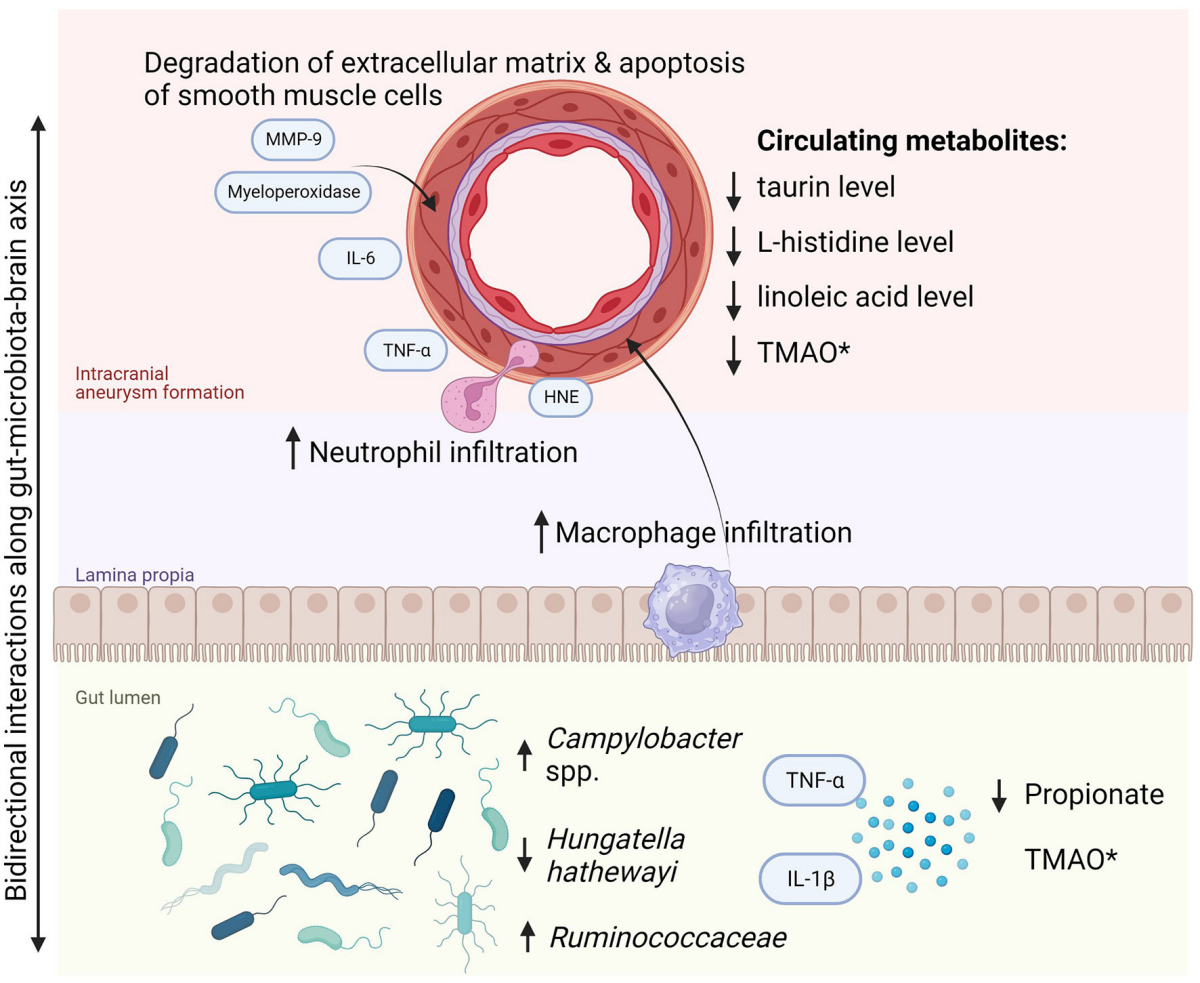
Pathway of intracranial aneurysm formation and progression due to gut dysbiosis. *Most cerebrovascular diseases are associated with increased TMAO in the gut. However, the reduced circulating TMAO level was noted in subjects with ruptured intracranial aneurysm and subarachnoid hemorrhage. HNE, human neutrophil elastase; IL, interleukins; MMP-9, matrix metalloproteinase-9; TMAO, trimethylamine N-oxide; TNF-α, tumor necrosis factor α.

The findings of this systematic review highlight an emerging understanding of the role of gut microbiota in intracranial aneurysm growth and rupture. There is also evidence of certain gut microbiota members discriminating symptomatic and asymptomatic aneurysms. Furthermore, it has been confirmed that gut microbiota plays a role in the pathophysiology of IA as demonstrated in an *in vivo* study with microbiota depletion (Shikata et al., 2019). The specific gut microbial composition might significantly diminish the risk of IA incidence and subsequently lower the probability of symptomatic manifestation. Two pathogens were identified to be crucial in the first aspect: *H. hathewayi* and *C. ureolyticus*. The former, a Gram-positive rod-shaped bacterium was first described in 2001 and classified as *Clostridium hathewayi*, then reclassified in 2014 to belong to the *Hungatella* genus (Kaur et al., 2014). It is an obligate anaerobe that symbiotically colonizes the human gut and in rare cases, it might be directly involved in bacteremia (Randazzo et al., 2015). In contrast, the intestinal abundance of *H. hathewayi* was associated with reduced inflammation and extracellular matrix remodeling in the cerebral arterial wall and with reduced metalloproteinase-mediated degradation of smooth muscle cells in cerebral vessels. These processes were consistent with diminished incidence of aneurysm formation and rupture rate. It is prudent to hypothesize that it has a causal relationship with the growth of UIA as it was proven in both discovery and validation cohorts, as well as in the animal model of *H. hathewayi* gavage in mice. However, its role in rupture and aneurysmal subarachnoid hemorrhage was only presented in the animal model with fecal transplant and bacterial gavage. Thus, it requires confirmation and validation in a clinical setting in human beings. As promising as these findings are, prior to their real-life application, they also must be reproduced in another independent study. Interestingly, *H. hathewayi* and *Campylobacter* spp. have not been found to play a role in any other cerebrovascular diseases (Zou et al., 2022). On the other hand, *Campylobacter* infection is linked to increased levels of interleukin-8, matrix metalloproteinase (MMP) 8, MMP-9, human neutrophil elastase, and myeloperoxidase, all of which were shown to be significant for formation and rupture of intracranial aneurysm (Nilsson et al., 2018; Kushamae et al., 2020). For a scheme of pathways of intracranial aneurysm formation and progression due to gut dysbiosis (see Figure 2). Overall, regarding the clinical application of the findings, we agree that the specific metagenomic signature of gut microbiota in subjects with cerebral aneurysms might potentially contribute to screening tests of blood and stool to non-invasively detect intracranial aneurysms or quantify the risk of rupture (Nowicki et al., 2023).

As for the symptomatic and asymptomatic IA, Ruminococcaceae (genera: *Fournierella, Ruthenibacterium*, and *Anaerotruncus*) and *Clostridiales*, along with Fusobacteria were found to play a role in the manifestation of aneurysm, with the latter one significantly diminished compared to asymptomatic patients. Indeed, the Ruminococcaceae family was found to also confer a risk for abdominal aneurysm and arterial stiffness (Menni et al., 2018; Ji et al., 2021). In addition, a study conducted by Xie et al. found that the *Ruminococcus* genus is linked to extracranial aneurysm size in rodents (Xie et al., 2020). The biological role of this genus has been associated with intrahepatic fat cumulation, thus possibly with lipid infiltration and consequently aneurysm rupture (Meir et al., 2021). The metabolic activity of microbiota was also found to be discriminative with regard to symptomatic manifestation. Among the symptomatic patients, propionate biosynthesis was found to be diminished, and peptidoglycan synthesis was elevated (Sun et al., 2022). Indeed, short-chain fatty acid propionate maintains microglial functions and might serve as a protective intervention against cerebrovascular damage secondary to hypertension (Hoyles et al., 2018; Bartolomaeus et al., 2019). Meanwhile, peptidoglycan, a well-known structure of bacterial cell wall has been historically linked to inflammation, which affects the vascular wall and contributes to aneurysm formation (Chalouhi et al., 2012).

Although to date there has not been a single study regarding the role of gut microbiota in cerebral vasospasm following aSAH, there is a strong rationale for conducting one. It has been shown that alteration of gut microbiome might lead to endothelial dysfunction which is one of the core mechanisms of vasospasm. Rustia et al. (2021) presented an experimental animal study showing that antibiotic treatment disrupting gut microbiota leads to endothelial dysfunction by the reduction of phosphorylated endothelial nitric oxide synthase (eNOS-p) to total eNOS ratio (a surrogate marker to eNOS activity). The gut dysbiosis obtained via a 3-week antibiotic regimen also produced increased spontaneous tone of the endothelium, decreased response to NG-Nitro-l-Arginine Methyl Ester (L-NAME) which is a non-selective nitric oxide synthase, and diminished ATP-mediated luminal dilation. Another contributing mechanism of vasospasm in gut dysbiosis might be the disruption of short-chain fatty acids (SCFA) expression. Moreover, fecal transplantation restores microbiome homeostasis, increases SCFA both in the gut and cerebral areas, and reduces neuroinflammation (Xiao et al., 2022). The plasma level of gut microbiota-dependent trimethylamine N-oxide (TMAO) in cerebral ischemia is usually elevated, while in aneurysmal subarachnoid hemorrhage, there was a reduced level in the only study conducted to date (Rexidamu et al., 2019; Schneider et al., 2020; Emonds et al., 2023). TMAO is a biomarker metabolite produced from phosphatidylcholine and choline. Due to its role in platelet responsiveness, it was found to correlate with ischemic cerebrovascular incidents. Its significance for cerebral vasospasm has not been established yet as the only study on TMAO in aneurysms presented with a small sample of vasospasm cases (*n* = 9) (Emonds et al., 2023). Thus, there is a strong demand for a study addressing the diversity of gut microbiota in post-aSAH vasospasm and delayed cerebral ischemia (see Section Future prospects).

## Possible interventions to alter microbiota to reduce the risk of aneurysmal growth and rupture

**Figure 3:**
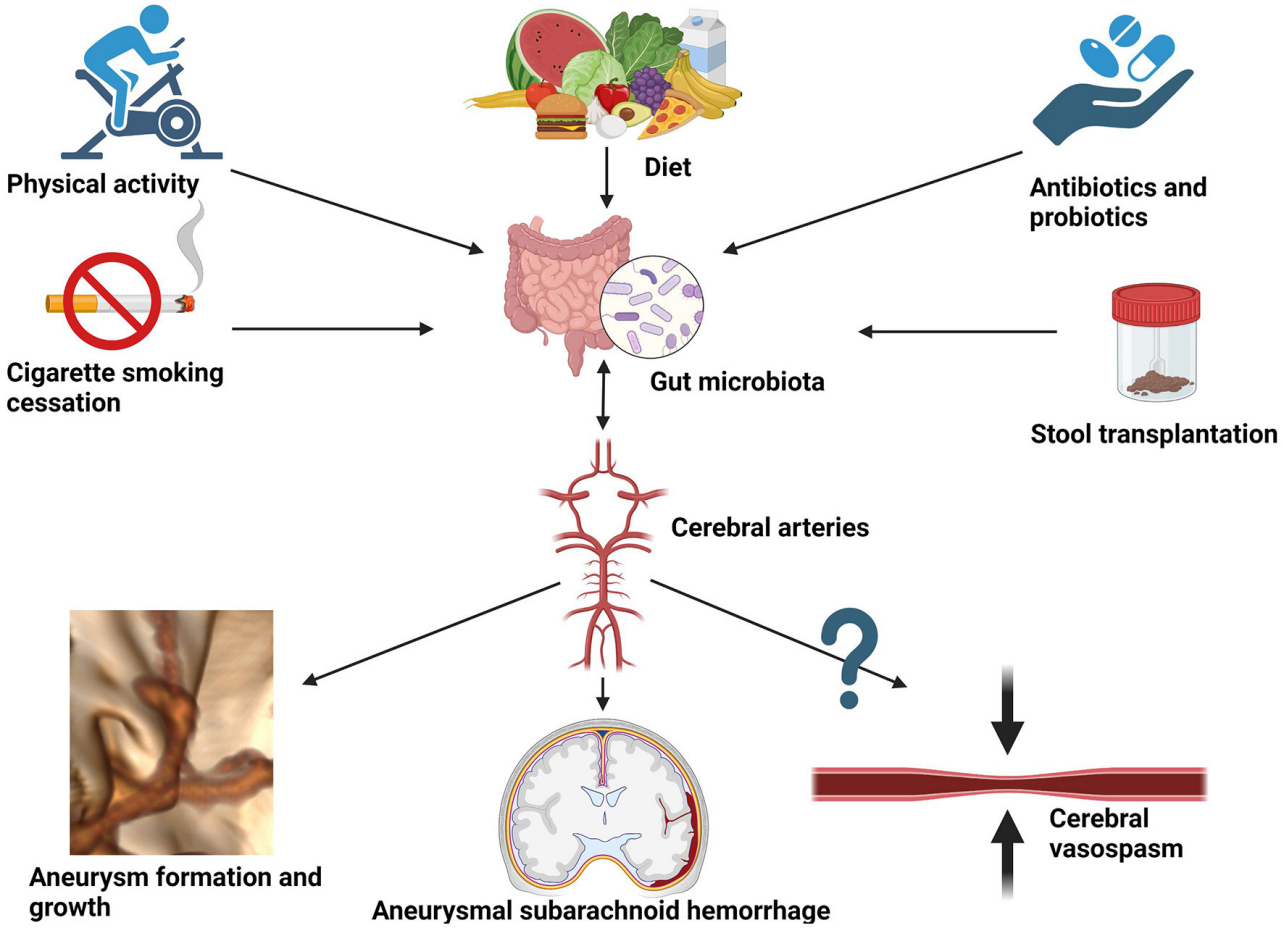
Potential environmental and interventional factors altering gut microbiota that could be considered for reducing the risk of aneurysmal growth, aneurysmal subarachnoid hemorrhage, and hypothetically also cerebral vasospasm.

The pathogenesis of intracranial aneurysms and subarachnoid hemorrhage is a multifactorial and complicated process that remains poorly understood. The importance of gut microbiota lies in mediating the immune process in the arterial wall of the forming aneurysm via the gut–brain–microbiota axis. Proinflammatory cytokines such as tumor necrosis factor α (TNF-α), interleukin-1, and interleukin-6 are associated with the destabilization of the aneurysmal wall and promote its rupture (Sathyan et al., 2015; Simon and Grote, 2021). As shown in this review, these fundamentals of neuroinflammation could potentially be altered by shifts in microbiota. In fact, environmental and interventional factors are known to impact the diversity of gut microbiota. Among others, they include dietary interventions, antibiotic or probiotic therapy, physical activity, or smoking cigarettes (see Figure 3).

Diet is a primary force affecting gut microbiota and its connection with intestinal taxonomic diversity is undisputed. Since the main reservoir for human campylobacteriosis is poultry, potential dietary interventions could focus on reducing daily chicken intake in favor of washed vegetables. Moreover, as cross-contamination is not uncommon during the industrial production of salads when handling raw chicken meat, public health interventions should encourage rigorous control of Campylobacteria in ready-to-eat salads and vegetable crops (Leifert et al., 2008; Habib et al., 2020). On the other hand, to obtain a relative abundance of *H. hathewayi*, dietary fiber containing barley β-glucan has been used in microbiomic research (Centanni et al., 2020). This way gut microbiota could be a potential target for the prevention and treatment of aneurysm growth and rupture via dietary interventions. As elegantly demonstrated by Li et al., taurine supplementation might have a protective role in IA formation (Li et al., 2020). Moreover, it could serve as a prognostic factor in subarachnoid hemorrhage (Pérez-Neri and Diéguez-Campa, 2018). Moreover, bioinformatics analysis performed by Liu H. et al., 2020 detected two genes, *CAV1* and *MYH11*, whose expression was downregulated in the intracranial aneurysm tissue (Liu H. J. et al., 2020). Furthermore, the expression was successfully upregulated by implementing probiotic treatment. The hypothetical model they presented combined publicly accessible datasets from Gene Expression Omnibus (GEO), including an animal study, and thus cannot be directly translated to clinical practice. Therefore, case–control studies on transcriptomics are necessary to compare gene expression in subjects with aneurysms and without, prior to, and after probiotic treatment. Other examples of interventional modalities to deliberately affect gut microbiota are cigarette smoking cessation (Gui et al., 2021), physical activity (Monda et al., 2017), the use of antibiotics (Shikata et al., 2019), and fecal transplantation from healthy donors (Figure 3). The latter has been shown to protect against intracranial aneurysm growth and rupture in the preclinical animal model (Li et al., 2020) and requires confirmation in a clinical setting. A comprehensive description of the influence of each factor on the gut microbiota extends beyond the scope of this review and can be appreciated in the following reference (Zou et al., 2022).

## Future prospects

As elegantly described elsewhere, the composition of the microbiota fluctuates on a constant basis, varying with age, season, gender, genetic factors of the host, environmental conditions, and lifestyle in general, with dietary habits playing a priority role (Hou et al., 2022). Thus, a question arises whether we can afford a definition of “normal microbiota” or “microbiota of a healthy person”. Moreover, are there any referential indicators for the intestinal ecosystem of a healthy person human being? According to the current state of knowledge, healthy microbiota composition is only a theoretical phenomenon, whereas in clinical settings, only a high diversity of gut microbiota in an individual might be considered healthy and robust (Manor et al., 2020). However, it is difficult to be objective because, for instance, intestinal transit time influences microbial composition to a great extent (Tottey et al., 2017). This brings us to a turning point in redefining the microbiota. While each of us may have a different composition of gut bacteria, microbial activity is almost identical. Therefore, the parameter to look out for when determining the “correctness” of the gut microbiota is its activity defined by the synthesis of metabolites relevant to physiological processes in the human body. Moreover, it is of high importance to consider differences in techniques used to analyze microbiota composition. Indeed, different variable regions vs. whole genome sequencing, different depth of sequencing, or different starters used might be responsible for the results obtained (Tourlousse et al., 2022). Thus, a consensus of the methodological approach seems to be of relevance to translate microbiota research into practice. Finally, such methodological guidelines would make meta-analyses feasible.

Without diminishing the validity of taxonomic approaches, our synthetic study proves that gut microbiota is definitely an ‘organ without organ’ as it affects the host's homeostasis and is of high potential as a target in individualized medicine. As a scientific community, we need to strive and put forth efforts to look for new microbiota-oriented diagnostic and/or therapeutic tools.

Due to the scarcity of research on the influence of microbiota on intracranial aneurysms and their complications, any further insight will be of value. The authors of this review are currently conducting a nationally funded (grant number 2021/41/N/NZ2/00844, National Science Centre, Poland) study on genetic, epigenetic, and microbiomic fundamentals in aneurysmal subarachnoid hemorrhage and cerebral vasospasm. In our study, we will evaluate gut microbiota in the feces of the subjects with subarachnoid hemorrhage, who are grouped according to the ultrasonographic status of their intracranial arteries (vasospasm or no vasospasm). The results might provide crucial data elucidating complex interactions along GMBA. Moreover, the results of the ongoing study will allow for the first multi-omics analysis in the field of cerebral aneurysms, searching for associations between genomics, epigenomics, and metagenomics.

Following the submission of this review, a third complementary wave of literature search took place to ensure the up-to-dateness of the study. We identified two articles that applied bioinformatic analysis to data from public repositories of subjects with IA. Due to this, we decided not to include these studies in the Section Results, where we depict data abstracted from observational studies. Instead, we here report the evidence generated by these publications. Ma et al. performed a Mendelian randomization study on a dataset from a genome-wide association study and detected one bacterial family and one genus in gut microbiota that reached the genome-wide statistically significant threshold. *Peptostreptococcaceae* (OR: 4.92; 95% CI: 1.32–18.32; *P* = 0.018) and *Streptococcus* (OR: 5.19; 95% CI: 1.25–21.56; *P* = 0.024) could increase the risk of IA (Ma et al., 2023). *Streptococcus* is known for mediating immune response via proinflammatory cytokines (interleukin-1 and interleukin-6), whereas *Peptostreptococcaceae* is involved in atherosclerosis and might promote the growth of aneurysms. Other bacteria that were linked to IA but did not reach GWAS significance were *Adlercreutzia, Clostridia, Rhodospirillaceae, Sutterella, Victivallis, Oscillospira*, and *Paraprevotella*. To confirm that any of these microbiota representatives might potentially serve as kind of a microbiological marker of the disease, observational studies are needed (Ma et al., 2023). Qin et al. performed a similar study utilizing the Mendelian randomization technique. They identified one family (*Porphyromonadaceae*) and two genera (*Bilophila* and *Ruminococcus*1) that might be a protective factor against IA. On the other hand, one family (*Streptococcaceae*) and two genera (Prevotella7 and Streptococcus) were associated with a higher risk of UIA (Qin et al., 2023). However, the single-nucleotide polymorphisms used in their study did not reach the genome-wide significance threshold (*p* < 5 × 10^−8^).

## Limitations and strengths

Although comprehensive and up to date, this review has several limitations. First, the pre-research protocol was not published. Although systematic review protocols are recommended to avoid unnecessary duplication, they are not routinely published as shown by the global survey (Tawfik et al., 2020). Second, the risk of bias analysis revealed that studies did not estimate sample size beforehand. This might have led to the downsizing of the samples and not producing all significant results, deviating from the scientific truth. Moreover, one publication presented the results of the preclinical study in the animal model, which limits the extrapolation of their conclusions. The most important strength of this study is its systematic nature and the fact that, to the best of our knowledge, it is the first such review in the literature to touch upon the relations between gut microbiota and intracranial aneurysms.

## Conclusion

Gut dysbiosis contributes to the development and rupture of intracranial aneurysms. There is also evidence that certain microbial fingerprints might be responsible for IA clinical manifestation. The proper gut abundance of *H. hathewayi* might protect against intracranial aneurysms and aSAH. On the other hand, *C. ureolyticus* might pose a risk of cerebral aneurysm rupture. Further research in a clinical setting is required to confirm the findings. There is an ongoing first study on cerebral vasospasm and metagenomics of the human gut microbiome. Dysbiosis of the gut microbiota is a potential target for prevention, early non-invasive detection, and treatment of cerebral aneurysms.

## Author contributions

TK, BP, and KS-Ż: data curation, formal analysis, investigation, methodology, and resources. TK and LS: funding acquisition. LS, KS-Ż, and ES: supervision and writing—review and editing. KS-Ż: validation. TK: writing—original draft. All authors contributed to the article and approved the submitted version.
